# Reflectance Spectroscopy for the Classification and Prediction of Pigments in Agronomic Crops

**DOI:** 10.3390/plants12122347

**Published:** 2023-06-16

**Authors:** Renan Falcioni, Werner Camargos Antunes, José Alexandre M. Demattê, Marcos Rafael Nanni

**Affiliations:** 1 Graduate Program in Agronomy, Department of Agronomy, State University of Maringá, Av. Colombo, 5790, Maringá 87020-900, PR, Brazil; wcantunes@uem.br (W.C.A.); mrnanni@uem.br (M.R.N.); 2 Department of Soil Science, Luiz de Queiroz College of Agriculture, University of São Paulo, Av. Pádua Dias, 11, Piracicaba 13418-260, SP, Brazil; jamdemat@usp.br

**Keywords:** C_3_ and C_4_ plants, digital technologies, high-throughput phenotyping, multivariate tools, pigment quantification, PLSR models, remote sensing

## Abstract

Reflectance spectroscopy, in combination with machine learning and artificial intelligence algorithms, is an effective method for classifying and predicting pigments and phenotyping in agronomic crops. This study aims to use hyperspectral data to develop a robust and precise method for the simultaneous evaluation of pigments, such as chlorophylls, carotenoids, anthocyanins, and flavonoids, in six agronomic crops: corn, sugarcane, coffee, canola, wheat, and tobacco. Our results demonstrate high classification accuracy and precision, with principal component analyses (PCAs)-linked clustering and a kappa coefficient analysis yielding results ranging from 92 to 100% in the ultraviolet–visible (UV–VIS) to near-infrared (NIR) to shortwave infrared (SWIR) bands. Predictive models based on partial least squares regression (PLSR) achieved R^2^ values ranging from 0.77 to 0.89 and ratio of performance to deviation (RPD) values over 2.1 for each pigment in C_3_ and C_4_ plants. The integration of pigment phenotyping methods with fifteen vegetation indices further improved accuracy, achieving values ranging from 60 to 100% across different full or range wavelength bands. The most responsive wavelengths were selected based on a cluster heatmap, β-loadings, weighted coefficients, and hyperspectral vegetation index (HVI) algorithms, thereby reinforcing the effectiveness of the generated models. Consequently, hyperspectral reflectance can serve as a rapid, precise, and accurate tool for evaluating agronomic crops, offering a promising alternative for monitoring and classification in integrated farming systems and traditional field production. It provides a non-destructive technique for the simultaneous evaluation of pigments in the most important agronomic plants.

## 1. Introduction

In recent years, corn, sugarcane, coffee, canola, wheat, and tobacco crops have garnered increased interest for plant and pigment phenotyping [1,2,3,4,5]. According to the FAO (2022), approximately 70% of global crop production comprises agronomic crops. Remote sensing techniques have proven to be effective in classifying crop varieties, cultivars, and genetics for phenotyping, providing efficient, accurate, and precise results worldwide [2,4].

Various techniques and equipment can be employed to optimize agricultural production strategies, according to Li et al. (2022) [6] and Wang et al. (2022) [7]. These include tools such as red–green–blue (RGB) sensors, multispectral image sensors (MSI), hyperspectral remote sensing (HRS), hyperspectral imaging sensors (HSI), and visible–near-infrared–shortwave infrared (VIS–NIR–SWIR) spectroscopy tools. These can be combined with machine learning and artificial intelligence algorithms [8]. This combination can lead to improved yields in a range of production methods, including indoor and vertical farming, as well as traditional agriculture [2,9].

The use of UV–VIS–NIR–SWIR spectra in a classification analysis, plant metabolism distinctions, and the assessment of growth, leaf structural–water interactions, and phenolic compounds has demonstrated potential as a valuable technique. The unique fingerprints that are present in agronomic crops allow for the effective classification of a large number of plant varieties, resulting in improved yields [1,2,3,4,5,10]. In this sense, the development of high-throughput phenotyping systems and plant pigment phenotyping can also be considered alternative tools of plant remote sensing.

Hyperspectral analysis, spanning ultraviolet (UV), visible, near-infrared (NIR), and shortwave infrared (SWIR) ranges, has emerged as a powerful tool in the realm of agriculture and plant sciences [2,11,12]. This technology leverages the unique spectral “fingerprints” of different plant species and conditions, thereby facilitating applications such as plant classification, growth assessments, and the monitoring of plant health and leaf structure–water characteristics. Such techniques have proven their efficacy in classifying diverse plant varieties, a factor that contributes significantly to improved horticulture crop yields [13,14]. With the ability to accurately identify and monitor specific plant varieties through their unique spectral signatures, farmers can implement more efficient practices, taking corrective action when spectral data suggest impending issues.

The precision of reflectance spectroscopy is further refined by selecting the most responsive wavelengths for analysis, given that different plant traits are more discernible at specific wavelengths [15,16,17]. This selection process is key to generating reliable and actionable data. However, it is essential to acknowledge that the success of these methods hinges on several factors. These include the quality of the spectral data, the accuracy of the classification algorithms employed, and the specific conditions in which the specific pigments are found in the leaves of plants [2,11,12]. Hence, while promising, the application of hyperspectral analysis demands careful consideration of these variables.

The Green Revolution of the 1960s, as well as the recent Digital Revolutions 4.0 and 5.0 [6,7], saw technological advancements applied to high-throughput analyses, contributing to an increase in food production for several agronomically important crops. This advancement allows decision-makers to utilize computational intelligence to enhance the classification, monitoring, and nutritional value of crops in fields, resulting in billions of dollars in annual economic benefits [13]. Consequently, artificial intelligence algorithms (AIAs) based on data mining (DM) [18], deep learning (DL) [7,19], and machine learning (ML) [20,21,22] present promising techniques for future non-invasive pigment analyses in crop sciences and remote sensing applications.

The application of remote sensing 5.0 techniques has seen an increase in efforts to estimate the use of machine learning, data mining, and deep learning to classify crops such as corn and soybean [23], wheat [6], coffee [24], and lettuce [14]. The use of UV–VIS–NIR–SWIR hyperspectral imaging is expected to be highly valuable [8,25]. Furthermore, the combination of the speed, precision, and accuracy [26,27,28] of hyperspectral equipment [23] coupled with the capacity of multivariate and artificial intelligence algorithm tools is crucial for pigment profiling crop modeling [5,6,7].

The aim of this study was to classify and predict the pigment phenotype of agronomic plants, such as corn, sugarcane, coffee, canola, wheat, and tobacco, through three main objectives. First, eight principal MLs and AIAs were used to discriminate these crops. Second, we examined the correlation of fifteen vegetation indices with pigment measurements. Finally, we simultaneously predicted the concentrations of chlorophyll, carotenoids, anthocyanins, and flavonoids based on the area and mass in leaves using partial least square root (PLSR) tools with UV–VIS–NIR–SWIR sensors in these plants.

## 2. Results

### 2.1. Descriptive and Variance Analysis-Based Attributes of Crops

**Figure 1:**
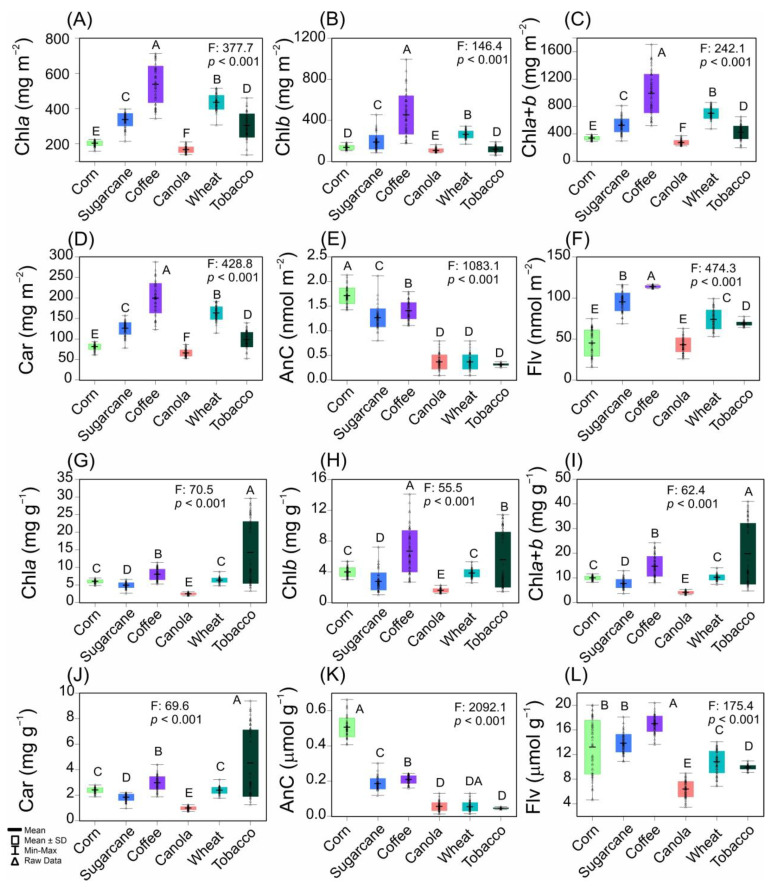
Boxplot of leaf pigment concentrations expressed by leaf (**A**–**F**) area and (**G**–**L**) mass from corn, sugarcane, coffee, canola, wheat, and tobacco. F-test by one–way ANOVA (*p* < 0.001). Different letters over the boxes indicate significant differences by Duncan’s test (*p* < 0.001) between crop plants. (*n* = 60). Dash: means; square: mean ± SD; outer spread: min–max; triangle: raw data. The abbreviations are described in Table S2.

The analyses of pigments revealed significant differences, with an F-value of approximately 146 at 1083 and a *p*-value of less than *p* < 0.001, as reported in Figure 1. The chlorophyll and carotenoid concentrations of coffee, wheat, and sugarcane presented a range of values from a minimum of 120 to a maximum of 990 mg m^−2^ (Figure 1A–D). Anthocyanins and flavonoids were detected in coffee, sugarcane, and corn, showing values between a minimum of 1.2 and a maximum of 1.8 nmol m^−2^ and between 78 and 118 nmol m^−2^, respectively (Figure 1E,F).

When pigment was expressed on a mass basis (Figure 1G–L), higher values were observed for coffee and tobacco in terms of the chlorophyll and carotenoid concentrations. Similarly, higher anthocyanin and flavonoid concentrations were observed in corn, sugarcane, and coffee (Figure 1K,L). We also analyzed leaf pigments based on both area and mass (Table S1), establishing minimum and maximum values for corn, sugarcane, coffee, canola, wheat, and tobacco (Figure 1 and Table S2). The twelve attributes analyzed demonstrated the coefficient of variation values spanning from 37.1% to 93.4%. All were classified as having high variance (Figure 1).

### 2.2. Hyperspectral Analysis in Leaves

**Figure 2:**
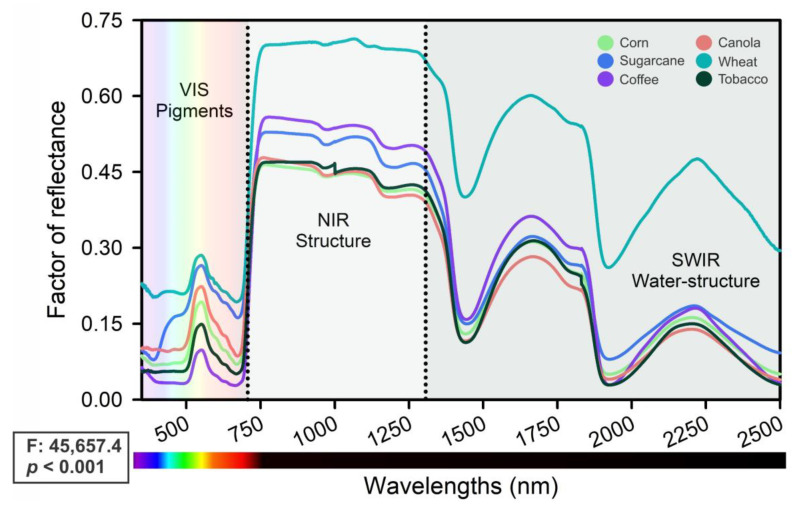
The reflectance factor was analyzed in expanded leaves from corn, sugarcane, coffee, canola, wheat, and tobacco. The spectral range included UV–VIS (350–700 nm, which shows pigments in the leaves), NIR (700–1300 nm, which reveals the structural components), and SWIR (1300–2500 nm, which represents the structural–water interactions). The dotted line indicates the inflection points at 700 and 1300 nm. One-way ANOVA F-test showed significance (*p* < 0.001) with 360 samples for 60 samples for each crop.

Figure 2 presents UV–VIS–NIR–SWIR hyperspectral curves for 360 of the total samples as corn, sugarcane, coffee, canola, wheat, and tobacco leaves. The permutation multivariate analysis of variance (F: 45,657.4; *p* < 0.001) reveals the wavelengths with the highest significance among all spectra. Variations in the reflectance factor were noted in the UV region (350–400 nm), where many phenolic and flavonoid compounds were observed, as well as in the visible region (400–700 nm), where leaf pigments such as anthocyanins, carotenoids, and chlorophylls were present.

The near-infrared region (700–1300 nm) showed structural differences in leaf tissues, such as pectin, hemicellulose, lignin, and cellulose, while the shortwave infrared (SWIR; 1300–2500 nm) was attributed to the structural water content of proteins and conjugate water in intrinsic structures, such as vacuoles and other organelles, as well as cell walls (Figure 2). The high variability (between 22.4 and 48.3%; 350–2500 nm) among the crop samples was primarily attributable to differences in pigments, structural composition, and leaf scattering (Figure 2).

### 2.3. Cluster Heatmap of Selected Wavelengths and Classification-Based UV–VIS–NIR–SWIR Bands

**Figure 3:**
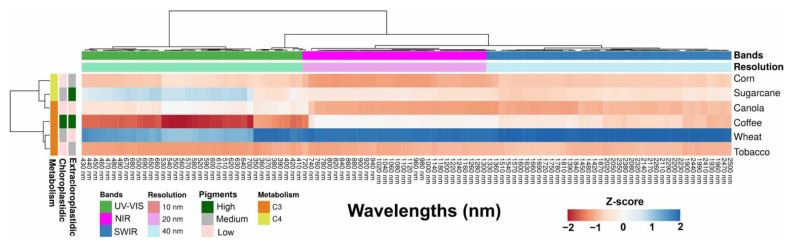
Cluster heatmap displayed the correlation between the spectral data of crops (corn, sugarcane, coffee, canola, wheat, and tobacco) and their pigments (chloroplastidic, such as chlorophylls and carotenoids, and extrachloroplastidics, such as anthocyanins and flavonoids). These are grouped by UV–VIS, NIR, and SWIR wavelength bands, and spectral resolution of 10, 20, and 40 nm. The color blue indicates a positive relationship between the spectral bands, pigments present in crop plants (C_3_ and C_4_ metabolism), and pigment concentrations, while red represents negative correlations, as per the Z-scores (*p* < 0.001).

Figure 3 displays a cluster heatmap created to visualize the relationship between spectral data and pigment concentrations. The association between hyperspectral values and pigment concentrations was leveraged to categorize pigments (chloroplast or extrachloroplast) and identify distinct crops. Blue colors represent higher reflectance signals for crops with substantial concentrations of chlorophylls (as they have two major peak absorptions, with blue peaks at 430 and 453 nm for chlorophyll *a* and *b*), and carotenoids (broad absorption range in blue (400–500 nm)), while deeper shades of green and red indicate crops with high anthocyanin and flavonoid concentrations. Lighter shades denote a lack of association between specific wavelength bands and a certain pigment concentration. Clustering patterns based on UV to VIS to NIR to SWIR bands and crop groups were discernible. The clustering revealed the dominance of one class of pigment or the interaction between reflectance and cell mesophyll scattering. For instance, most UV–VIS bands exhibited similar correlation patterns within their own group (C_3_ vs. C_4_ metabolism; Figure 3).

The NIR or SWIR bands demonstrated a strong and negative correlation with specific wavelength values, suggesting an interaction between pigments and leaf thickness, as well as the occurrence of scattering phenomena within leaf structures such as cell parenchyma and intercellular spaces. Nevertheless, carotenoids (Car), anthocyanins (AnC), and flavonoids (Flv) showed no correlation with the NIR–SWIR-linked spectra associated with the structure and structure–water of the bands. All wheat samples exhibited a positive Z-score across all bands from UV–VIS to sugarcane. Furthermore, the clustering of pigments and metabolism reveals that each crop plant possesses a specific fingerprint, which could potentially be associated with a specific vegetation index. This association takes into account the vegetative growth stages, and whether they are associated with UV–VIS bands (350–700 nm), NIR bands (700–1300 nm), or SWIR fingerprints (1300–2500 nm).

### 2.4. Principal Component Analysis (PCA), Correlation Coefficients, and Loadings of the Wavelengths

**Figure 4:**
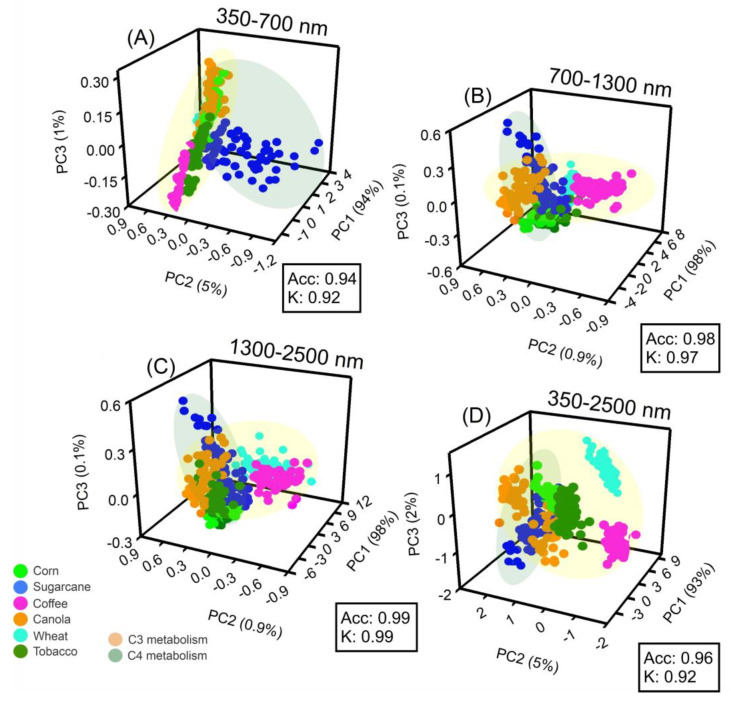
Three–dimensional plot for the first PC1, PC2, and PC3 of the spectroscopy data; (**A**) 350 at 700 nm; (**B**) 700 at 1300 nm; (**C**) 1300 at 2500 nm; (**D**) 350 at 2500 nm. Data were collected for hyperspectral data from corn, sugarcane, coffee, canola, wheat, and tobacco plants. Orange and green colors display correlation clusters of C_3_ or C_4_ metabolism-based model phenotyping for similar groups in crops. Acc represents accuracy, and K represents the kappa coefficient. (*n* = 60).

The first three principal components (PCs) accounted for 100% of the variance in the six spectral analyses (UV–VIS–NIR–SWIR) of corn, sugarcane, coffee, canola, wheat, and tobacco crops. Based on the PCA 3D plot in Figure 4, two primary clusters formed between C_3_ and C_4_ metabolisms (350–700 nm, 700–1300 nm, 1300–2500 nm, or 350–2500 nm; Figure 4A–D). The high precision of the results was demonstrated by the accuracy and Kappa coefficients, which were approximately greater than 0.94 (Acc) and 0.92 (K), respectively (Figure 4A–D).

**Figure 5:**
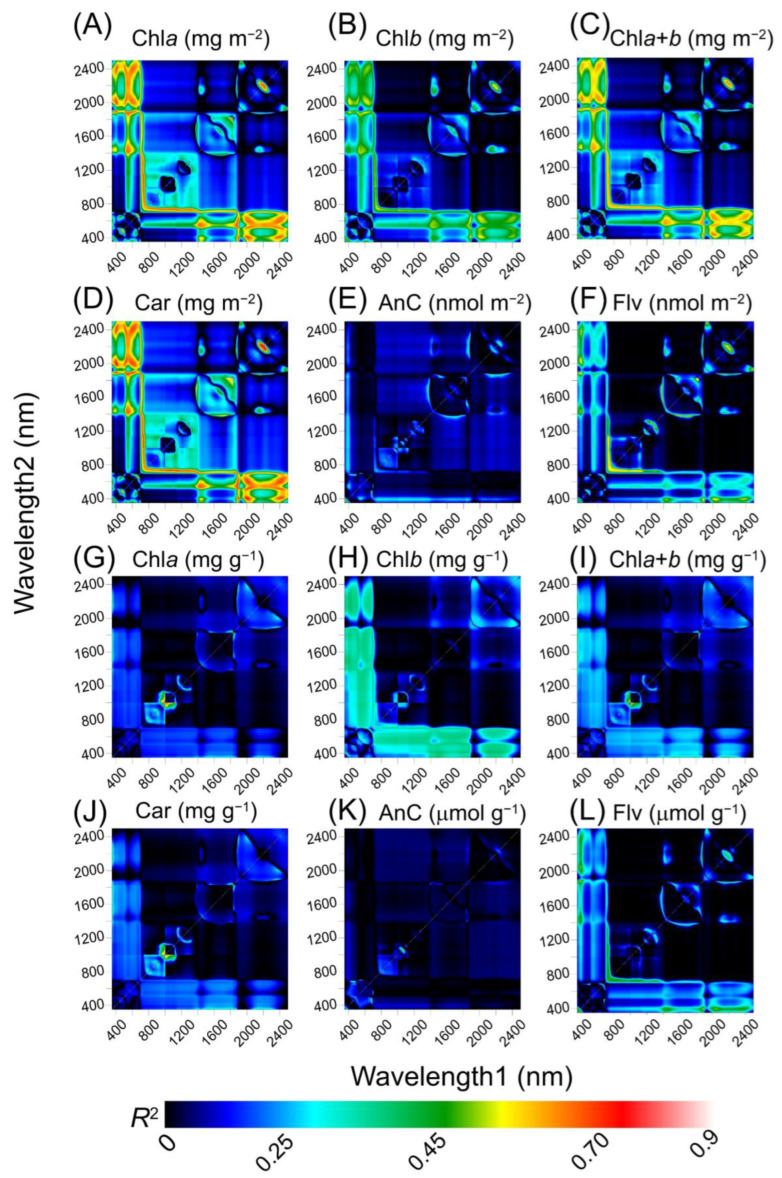
Count plot map of the coefficient of correlation (R^2^) from the linear regression between pigments and wavelength1 vs. wavelength2 for 350 to 2500 nm, by pigments expressed in based (**A**–**F**) area and (**G**–**L**) mass. Dark to light red displayed increased associations.

The correlation coefficients (CCs), principal component loadings (PCLs), and hyperspectral vegetation index (HVI) were obtained from the principal component analysis (PCA) across different crops. The results reveal the presence of three PCs, CC, HVI, and PCL, for the majority of the six spectral datasets (Figure 5 and Figure S1A,B). Visible (VIS) wavelengths were the dominant contributors to the first PC, which was linked to 555 and 660 nm. Near-infrared (NIR) wavelengths dominated the second PC, which was linked to 710, 940, 1080, and 1190 nm, with minor effects on the CC. Shortwave infrared (SWIR) wavelengths were the major contributors to the third PC, which combined the HVI, CC, and PCL with peaks and valleys at 1470, 1850, and 2245 nm.

A high correlation between NIR and SWIR was confirmed in the CC-associated PC1 (Figure 5 and Figure S1). The shapes of the HVI and PCL were complex, represented multiple contributions from UV–VIS–NIR–SWIR bands (Figure 5 and Figure S1B), and showed a non-random distribution in the pigment phenotyping-based area and mass (Figure 5A–L).

### 2.5. Machine Learning and Artificial Intelligence Algorithms for Classification

**Figure 6:**
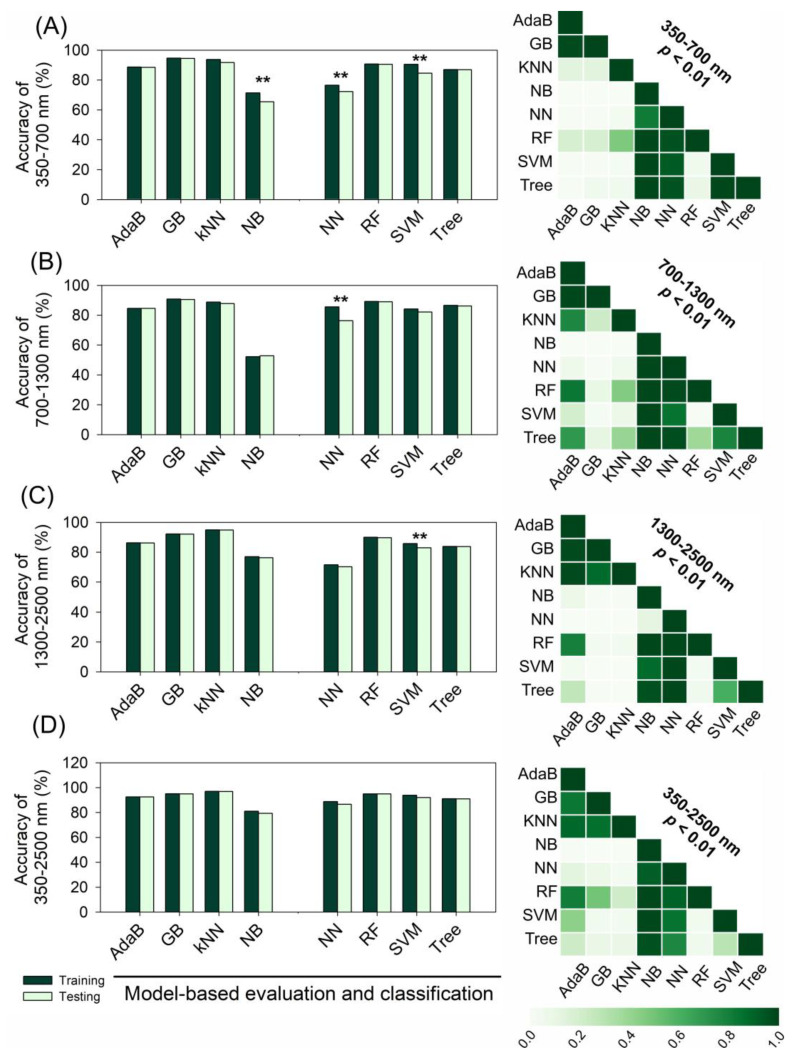
The accuracy of training and testing for various ML and AI algorithms, including adaboost (AdaB), gradient boosting (GB), K-nearest neighbors (KNN), naive bayes (NB), neural network (NN), random forest (RF), support vector machines (SVM), and tree (Tree), on hyperspectral data of crops. (**A**) Accuracy for the range of 350–700 nm. (**B**) Accuracy for the range of 700–1300 nm. (**C**) Accuracy for the range of 1300–2500 nm. (**D**) Accuracy for the entire range of 350–2500 nm. Asterisks indicate significant differences (*p* < 0.01) according to Student’s *t*-test. The Pearson’s correlation matrix on the right displays the probabilities of the scores for each model being higher than the scores for the models in the column. Small numbers indicate the probability of negligible differences.

Phenotypic characterization of the pigments in corn, sugarcane, coffee, canola, wheat, and tobacco was performed using UV–VIS–NIR–SWIR hyperspectral data and ML and AI algorithms (Figure 6). Eight algorithms were employed, namely, adaboost (AdaB), gradient boosting (GB), K-nearest neighbors (KNN), naive bayes (NB), neural network (NN), random forest (RF), support vector machine (SVM), and tree. These algorithms displayed a range of performances in classifying crops. Cross-validation data were used to classify crops using UV–VIS–NIR–SWIR spectra (Figure 6A–D).

The NB algorithm demonstrated lower accuracy than the other AI algorithms, and the NN displayed a lower correlation with the accurate crop classification produced by the other AI algorithms. However, the AdaB, GB, KNN, NB, NN, RF, SVM, and Tree algorithms all achieved ≈100% accuracy with high precision and efficiency. The SWIR bands showed higher accuracy and precision and a faster evaluation and confusion matrix generation than the UV–VIS or NIR bands (Figure S2 and Figure S3).

For example, NB, NN, and SVM demonstrated moderate accuracy and precision in the classification of crops within the ranges of 350–700 nm and 700–1300 nm, with 40–76% accuracy and precision in classifying crops. The use of the full spectra with individual AI algorithms demonstrated high accuracy, low error, and high precision in crop classification (Figure S2 and Figure S3). The results indicate significant discrimination (*p* < 0.01) in high-throughput phenotyping using individual AI algorithms for the UV–VIS, NIR, and SWIR bands (350–2500 nm).

### 2.6. Calibration, Cross-Validation, and Prediction Simultaneous Models by Crop Leaf-Based Partial Least Squares Regression

The results of spectral assessment for all crops during the mode calibration, cross-validation, and prediction steps are presented in Table 1 and Table S3, and Figure S3. The optimal number of components selected was four PCA–PLSR factors, determined through leave-one-out cross-validation. The correlation coefficients (R^2^) at the calibration phase were ≥0.77 and reached a maximum of 0.89, which is similar to the results for the cross-validation phase (≥0.76, max. 0.88) and the prediction phase (≥0.66, max. 0.89). Additionally, high values of offset, root mean square error (RMSE), and RPD (≥2.1, max. 3.0) were observed, indicating significance in all PLSR parameters. The models showed low or approximately zero bias, demonstrating the absence of bias for corn, sugarcane, coffee, canola, wheat, and tobacco plants.

In general, it is emphasized that increasing the number of selected target-specific wavelength regions for cross-validation and prediction models can lead to high accuracy (R^2^ > 0.77), as shown in Figure S3. For instance, the partial least squares regression (PLSR) method based on UV–VIS–NIR–SWIR hyperspectral data predicts β-loadings and weighted coefficients. However, compared to models that predict pigments in area and mass contents, the reflectance factors in the UV–VIS, NIR, and SWIR regions show high amplitudes of difference coefficients and distinct loadings, with great differences being observed.

To achieve higher precision than the models reported in Table 1 and Figure S3, it is important to consider many peaks and valleys distributed in all spectra. In this way, the high-throughput phenotyping crop-based pigments and β-loadings and weighted coefficients predicted by the PLSR method display larger differences in shape, although they are complex and represent several scattering contributions. Nevertheless, here, the generated models are of high accuracy and precision and have minimal bias and noise.

### 2.7. Vegetation Indices and Pigment Profiling

**Figure 7:**
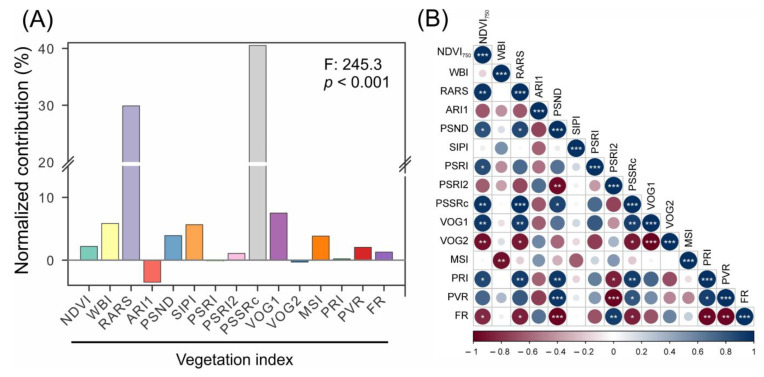
Model-based UV–VIS–NIR–SWIR reflectance hyperspectral data for simultaneous classification of corn, sugarcane, coffee, canola, wheat, and tobacco crops. (**A**) shows the relative vegetation index. (**B**) Pearson’s correlation (ranging from −1 to +1, with white asterisk for * *p* < 0.05; ** *p* < 0.01; *** *p* < 0.001). Red to blue scale indicate negative and positive correlations, respectively. The thickness of the line indicates the strength of the correlation. Abbreviations for the models are defined in Table S1.

Combining vegetation indices (VIs) resulted in a total of fifteen VIs, which showed both positive (13) and negative (2) statistical values for the classification and estimation of crop attributes (Figure 7 and Figure S3; Table S3). The optimal band combinations for VIs were determined to possess high accuracy, precision, and significance (F: 245.3; *p* < 0.001) for classifying, predicting, and monitoring pigments. The most responsive indices, PSSRc and RARS, demonstrated high accuracy in distinguishing all phenotypes utilizing UV–VIS–NIR–SWIR hyperspectral analyses (Figure 7A). The correlation between VIs was evaluated using the circular correlation coefficient graph and showed high positive and negative correlations ranging from −1 to +1 (*p* < 0.001) (Figure 7B). NDVI_750_, RARS, PSND, and PSSRc displayed strong positive interactions, while PSRI2 and FR showed strong negative interactions (Figure 7B). SIPI, WBI, and MSI demonstrated minimal or negligible correlations with other VIs (Figure 7B).

**Figure 8:**
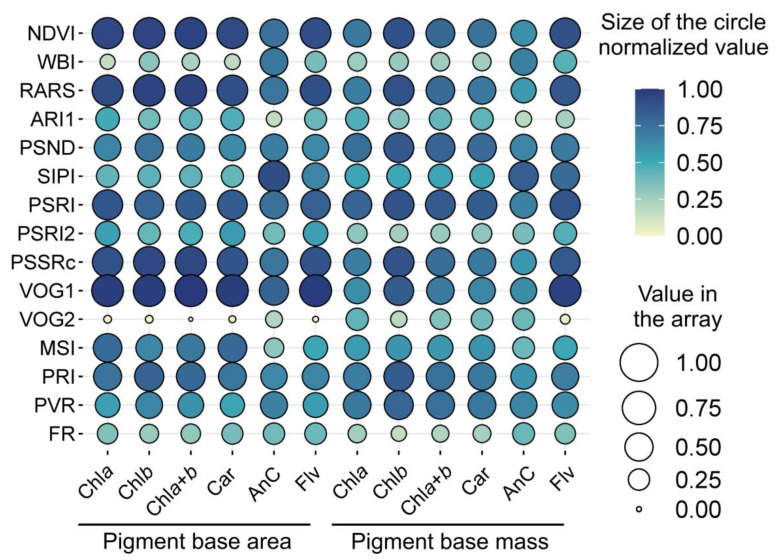
The balloon plot displays the normalized values to predictions of vegetation indices and pigments. The color and size of the circles indicate the reliability of the predictions. The names of the vegetation indices are shown on the left, and the site names for the predicted pigments, expressed as base area and mass, are displayed at the bottom. The relationship is indicated by a transition from yellow to blue, where yellow represents low values and blue represents high values. The size of the circles represents the association between the analyzed attributes, where smaller circles indicate weaker associations and larger circles indicate stronger associations.

Figure 8 displays the results of the combination of individual vegetation indices with pigment concentrations by both the area and mass. The prediction analysis showed that WBI, ARI, PSRI2, VOG2, and FR had limited correlations and associations with chlorophylls, carotenoids, anthocyanins, and flavonoids (correlation normalized below 0.50). These findings suggest that the selected reflectance indices have high potential for accurate and precise classification and prediction for the six crops analyzed. On the other hand, other indices showed higher correlation coefficients (above 0.5), and a significant portion of the scatter points fell within the 99% confidence interval of prediction, indicating the suitability of all models tested for each crop.

## 3. Discussion

### 3.1. Remote Sensing Sensor and Pigment Phenotyping in Leaves for High-Throughput Monitoring Crops

The application of phenotyping for crop-based pigment profiling through remote sensing techniques shows substantial promise. The use of full spectra based on hyperspectral curves has been demonstrated to provide a more precise classification and estimation of pigment profiles in C_3_ and C_4_ plant metabolisms compared to range spectra [3,11,29]. Moreover, the UV–VIS–NIR–SWIR wavelength bands and sensor resolutions contribute significantly to the efficient differentiation of crop metabolism (C_3_, C_4_, or CAM) [7,8,16,20]. Multivariate PCA has also been utilized with high accuracy and precision to differentiate C_4_ metabolism (as in corn and sugarcane) from C_3_ metabolism (as in coffee, canola, wheat, and tobacco) using select UV–VIS to NIR to SWIR bands.

The potential for high-throughput pigment phenotyping to evaluate and track growth and development, biophysical and biochemical characteristics, and diseases in crop production has been demonstrated in recent studies [1,2,3,8,13]. Spectral variations in pigment concentrations, including chlorophyll *a* and *b*, are closely correlated with differences in agronomic traits, such as plant height, grain yield, growth cycle, photosynthesis, transpiration, and water use efficiency [6,23,30,31].

Integrating hyperspectral sensors with chemometric techniques has proven effective for identifying and predicting a range of crop characteristics. For example, many studies have demonstrated the use of hyperspectral analyses to differentiate between livestock-integrated farming systems and indoor and vertical farming productions and to determine crop leaf characteristics [6,32,33,34]. Thus, our first and second objective proposed methods have also shown potential for increasing yield production in crops such as wheat and canola.

According to recent studies by da Silva Junior et al. (2018) [35] and Wang et al. (2022) [7], biosensors are the most promising tools for remote sensing, as demonstrated in Figure 1, Figure 2, Figure 3, Figure 4 and Figure 5. Hyperspectral analysis of chlorophylls and carotenoids in the VIS band has proven useful for monitoring [13,36]. Meanwhile, the best analysis of anthocyanins and flavonoids can be performed along with other pigment classes in the UV–VIS band [7,37,38]. This approach is a better tool for monitoring the status of six crops, and the increased presence of bioactive compounds or antioxidants in crops holds promise for increased yields [8,13,39,40]. Therefore, the UV–VIS band display serves as a robust technique for phenotyping and chemometrics, amalgamating numerous high-performance attributes in crop sciences, such as rapid precision, minimal sample requirement, and transparent technology for sample analysis, while safeguarding human health, safety, and quality [16,40].

### 3.2. Artificial Intelligence Algorithms Improvement Selection Pigment in Crops

The integration of remote sensing with AI techniques has proven highly effective for crop phenotyping and has contributed to improvements in crop yield, disease resistance, and cultivar selection. The use of hyperspectral analyses in conjunction with AI algorithms, such as random forest (RF) and neural network (NN), for pigment-based profiling has proven effective for classifying and monitoring six key crops with high accuracy. These algorithms can effectively link hyperspectral data with various factors, including nutritional deficiencies, heat and cold stresses, and crop yield [8,34,41].

Accordingly, the most accurate algorithms identified in previous studies include AdaBoost, gradient boosting, k-nearest neighbors, naive bayes, neural networks, random forests, support vector machines, and decision trees [7,21,28]. However, it is important to note that an accuracy greater than 60% does not necessarily guarantee a strong correlation with pigment concentration in C_3_ and C_4_ plants. The use of AI algorithms offers promising prospects for improving the extraction of complex interactions between hyperspectral data and pigment complexes, resulting in more accurate spectral data classification compared to other spectroscopic techniques. However, further research is needed to understand why AI algorithms do not respond to certain changes in the growth and development stages of C_3_ and C_4_ plants. Thus, our first and second objectives proposed in this method for our analysis are as follows.

### 3.3. Quantitative and Optimization PLSR Models to Estimate Pigments in Crops

In recent studies by Zhou et al. (2022) [38] and Zhang et al. (2022) [2], PLSR models were utilized to quantify the correlation between ultraviolet, visible, infrared, and short-infrared spectral data and pigment concentration data in six crops: corn, sugarcane, coffee, canola, wheat, and tobacco. The data were divided into calibration (70%) and validation (30%) sets, with the calibration set of 270 samples and the validation set of 90 samples achieving high accuracy and precision [4,42]. The results highlighted robust generation models based on R^2^, offset, RMSE, RPD, bias, and weight coefficients, although estimating anthocyanins (AnC) and flavonoids (Flv) proved more challenging [40,43]. Despite the variation between C_3_ and C_4_ plants, the highest prediction values were obtained for AnC and Flv, with a full spectra method applied to crop analysis [13,34]. Therefore, spectral data pre-processing was found to remove irrelevant information and improve robustness and accuracy [23,44], but the results here show that pre-processing may not be necessary, as spectral data without pre-processing improved the accuracy, precision, and reliability of PLSR models [13,44].

PLSR models require an optimal four factors [13,16], but without evidence of overfitting based on all parameters tested (Table 1). This work is significant, demonstrating the wide application of the PLSR method for detecting issues in future field crops using remote sensing and biosensors within integrated farming systems, as few studies have used combined UV–VIS–NIR–SWIR hyperspectroscopy to analyze Chls, Car, AnC, and Flv pigments in the leaves of C_3_ and C_4_ agronomic plants [2,40]. In this way, combining several types of sensors and conducting diverse studies on the large number of parameters of many plants at high speed can additionally be used for the estimation of plant morphological parameters, physiological processes, and biochemical composition. Therefore, the use of reflectance spectroscopy for the classification and prediction of pigment profiling in agronomic crops and for other plant research could be an effective strategy for optimizing growing conditions. This could contribute significantly to progress in the field of agronomic research and practice and relate to our thirty objectives.

The hyperspectral vegetation index (HVI) is emerging as a vital tool for plant phenotyping, particularly for assessing pigmentation levels such as chlorophyll *a*, *b*, *a*+*b*, carotenoids, anthocyanins (AnC), and flavonoids (Flv). Utilizing reflectance hyperspectroscopy, HVI can select the most responsive wavelengths, optimizing methods for remote and proximal sensing of physiological, biochemical, and morphological characteristics of plants. This provides a non-invasive, highly accurate method for assessing plant health and development, and it could revolutionize current agronomic practices. As such, HVI presents a promising alternative for enhancing our understanding and management of pigment phenotyping in plants. In this sense, using a specific wavelength, such as 435, 470, 550, 680, 685, 705, or 750, instead of broader bands such as blue, green, red, or near-infrared, holds more promise in characterizing C_3_ and C_4_ crops. Therefore, HVI algorithms and vegetation indices based on narrow bands could potentially enable more precise classification when there are different pigments present in crop varieties within the same environment.

A recent study by Koh et al. (2022) [45] discussed how hyperspectral vegetation indices (VIs) are increasingly being used in agriculture and plant phenotyping to estimate plant biophysical and biochemical traits. This study presented an automated hyperspectral vegetation index (AutoVI) system for the rapid generation of novel trait-specific indices and showed that AutoVI can rapidly generate complex novel VIs that correlate strongly with the measured chlorophyll and sugar contents in wheat [45,46].

While many reflectance indices have demonstrated superior performance in estimating certain plant traits compared to existing vegetation indices [47], there is still a need for a more robust model [15,48]. Specifically, we lack a system that adequately incorporates data related to anthocyanins and flavonoids in plants of agronomic importance [43,48]. Enhancing the current models to incorporate these pigment compounds could provide a more comprehensive assessment of plant health and development, thus further optimizing high-throughput plant pigment phenotyping platforms [36,41,47]. Therefore, it is crucial to continue research efforts to enhance the accuracy and efficiency of plant pigment phenotyping methodologies.

### 3.4. Vegetation Indices Combined for Pigment Phenotyping

Vegetation indices (VIs) are utilized for quantifying and expressing variables in crops, addressing the phenotyping gap discussed in numerous studies [16,34,47,49]. VIs, obtained by combining remote sensing reflectance data from UV–VIS–NIR–SWIR regions, are simple and effective parameters for characterizing vegetation cover and plant growth status. Combining reflectance indices with machine learning (ML) algorithms has led to successful phenotyping with high accuracy and precision [16,34]. Thus, the relative contributions of VIs were studied and could be used to select VIs corresponding to each pigment in different crops and for successful monitoring [24,50].

Morphological and anatomical changes can be identified using different VIs [7,13,16]. Evaluations of the mutual effect of VIs and pigment biosynthesis and degradation showed that specific or range bands can correlate with pigment classes and concentrations, structural components, and cell organelles. Digital agriculture tools linked with high-throughput pigment phenotyping can classify changes in C_3_ and C_4_ plant production features with high accuracy, speed, and efficiency at a low cost per sample [2,7,34,49]. These changes facilitate pigment profiling and identification by hyperspectral sensors, machine learning, AIAs, and recent HVI algorithms, providing an alternative to the standard approach of distribution within the leaf profile, changing both the optical properties of leaves in visible bands (>500 nm) and generating different VIs.

Our results align with those of Fu et al. (2021) [4] and Braga et al. (2021) [29], in which pigment concentrations and contents represent at most one responsive pathway for the classification of distinct plants [4,29]. Thus, the initial hypothesis indicated that the monitoring status significantly impacts classification, but the estimation process requires monitoring over a different timescale. This is confirmed when significant alterations in the visible and near-infrared spectra are not correlated with classic Vis, such as NDVI_750_, VOG, PSI, and PRI, despite extensive changes in colorimetric patterns and leaf reflectance factors (R) (Figure 2, Figure 3 and Figure 4). It is noteworthy that changes in cellular components should not affect spectral changes in the visible spectrum [13], but this phenomenon is commonly observed both in ranges and in full bands [47]. Therefore, the correlation previously reported is valid, as are the hypotheses initially described.

The internal structure of a leaf, such as the volume of mesophyll cells, varies among species [29]. As a result, the near-infrared (NIR) and shortwave infrared (SWIR) regions are greatly influenced by air cell interfaces. Additionally, the outer surface characteristics, including waxiness and epicuticular metabolites, trichomes, and stomates, also impact reflectance spectra in important crops. Therefore, the interplay of these external and internal leaf features with reflectance factors and pigment phenotyping may alter the energy flow within the leaf, potentially inducing toxic effects and impeding photosynthesis. Hence, the use of remote sensing sensors for high-throughput pigment phenotyping in crops is of utmost importance.

In contrast with Crusiol et al. (2022) [51] and Crusiol et al. (2023) [23], who did not find significant variation in pigments or the water status in soybean crops when monitored by combined AIAs, NB, and remote sensors, our study observed this variation in energy dissipation components. These components are mostly influenced by non-photosynthetic pigments, thermal influence by antioxidant mechanisms, the efficient use of water (WUE), and leaf water content (WBI, DSWI–5) (Figure 7 and Figure 8). Therefore, these components exhibited changes in VIs across all regions of the analyzed spectra (350–2500 nm) [13]. Thus, it is important to evaluate the impact of grafting and various classes of pigments on crops such as corn, sugarcane, coffee, canola, wheat, and tobacco. We found that plants subjected to these classifications exhibited a higher degree of precision and accuracy in their monitoring status. This precision and accuracy were superior to, for instance, those stemming from integrated systems of cultivation and digital agriculture.

## 4. Material and Methods

### 4.1. Plant Materials

Six plants of agronomic importance, corn (*Zea mays* L.), sugarcane (*Saccharum officinarum* L.), coffee (*Coffea arabica* L.), canola (*Brassica napus* L.), wheat (*Triticum aestivum* L.), and tobacco (*Nicotiana tabacum* L.), were collected from the Plant Cultivation Farms of the State University of Maringá (Maringá, Paraná, Brazil). The selection of these crop plants was based on their leaf development patterns. A total of 60 leaves were collected from each plant group, resulting in a total of 360 samples analyzed for modeling. Furthermore, plant metabolism was clustered into C_3_ and C_4_ categories based on wavelength bands, with corn and sugarcane classified as C_4_ metabolism, and coffee, canola, wheat, and tobacco classified as C_3_ metabolism. All leaves were collected and immediately taken to the laboratory for in vivo analyses (hyperspectral measures) and in vitro analysis (extraction of pigment profiles) during the vegetative growth phase. For leaves of the wheat plants, the flag leaf was evaluated.

### 4.2. Pigment Quantifications and Hyperspectral Analysis

The quantification of chlorophylls and carotenoids (Chl*a*, Chl*b*, Chl*a*+*b*, and Car), anthocyanins (AnC), and flavonoids (Flv) was performed using a methanolic extract-based method. The absorbance curve of pigments in vitro was analyzed between 350 and 1100 nm using a Shimadzu UV–3600 Plus UV–VIS–NIR spectrophotometer (Shimadzu Inc., Tokyo, Japan). The quantification of pigment profiles was performed and expressed in terms of area and mass, as detailed in [14,26].

In addition, hyperspectral reflectance was measured using spectroradiometers (ASD Inc; FieldSpec 3, Boulder, CO, USA) across the ultraviolet, visible, near-infrared, and shortwave infrared bands, following the methodology outlined in [15]. In brief, the PlantProbe^®^ leaf clip (Analytical Spectral Devices ASD Inc., Longmont, CO, USA) was used to ensure data acquisition free of atmospheric effects. Standard white reference plates (Spectralon^®^, Labsphere Inc., Longmont, CO, USA) were employed for equipment calibration and optimization. Reflectance spectra of the leaves were obtained in the 350–2500 nm range. The equipment was programmed to perform 50 readings for each sample, generating an average spectral curve. Measurements were taken at a single point on the adaxial face of the leaves. Furthermore, the same leaves used for pigment quantification were also analyzed using spectroradiometers to establish correlations between the pigment profiles and the corresponding reflectance spectra. This step was crucial for generating and validating the models.

### 4.3. Statistical Analyses

#### 4.3.1. Analysis of Variance and Descriptive Statistics

One-way analysis of variance was performed to analyze the data. The results were considered statistically significant if the *p*-value was less than 0.001. To compare attributes, a post hoc Duncan’s test was applied. Pearson’s correlation was also calculated (*p* < 0.001). The results included the means, medians, minimum, maximum, and coefficient of variation (CV) of the calculated data, following the methods reported by [27].

#### 4.3.2. Analysis of Leaf Reflectance Spectral Fingerprints

Based on the hyperspectral reflectance curves, parameters derived from the machine learning and artificial intelligence algorithm decision analysis were evaluated for the main fingerprint groups associated with wavelengths (Table S1). The fingerprints, along with vibrational modes, were related to the C_3_ and C_4_ metabolisms of corn, sugarcane, coffee, canola, wheat, and tobacco crops. The correlations of coefficients, principal component (PC) loadings, beta-loadings, and weighted coefficients were analyzed to identify the key fingerprints. Principal component analysis (PCA) was performed using The Unscrambler (CAMO AS, Oslo, Norway).

#### 4.3.3. Machine Learning, Artificial Algorithms, and Hyperspectral Vegetation Index

Machine learning (ML) and artificial algorithms (AIAs) were utilized to perform routine analysis using Orange software scripts. The algorithms employed included adaboost (AdaB), gradient boosting (GB), kernel K nearest neighbors (KNN), naive bayes (NB), neural network (NN), random forest (RF), support vector machines (SVM), and tree (Tree). The evaluation model was based on a 70:30 data split, with 70% of the data used for training and 30% for testing. The performance of the prediction AIAs was evaluated in terms of rank-performed precision and recall data, as detailed by [28]. In addition, each algorithm model was subjected to a confusion matrix analysis to assess the predicted data. All algorithm testing followed [13]. In addition, the Hyperspectral Vegetation Index (HVI) method was used to calculate all possible combinations of two spectral bands, each corresponding to a unique HVI algorithms. These HVIs are then correlated with leaf optical property efficiency measures, using the Pearson correlation coefficient and coefficient of determination, and visualized as contour maps [23].

#### 4.3.4. Vegetation Indices

The vegetation indices (VIs) were calculated based on the descriptions in Table S1. Reflectance hyperspectral data were tested with 15 VIs, such as NDVI_750_, WBI, RARS, ARI1, PSND, SIPI, PSRI, PSRI2, PSSRc, VOG1, VOG2, MSI, PRI, PVR, and FR (Table S1). These VIs were used to correlate pigment profiling and make decisions for the best crop classification based on correlation and association with a significance level of *p* < 0.001.

## 5. Conclusions

In conclusion, this method enables efficient and simultaneous quantification of chlorophyll, carotenoids, anthocyanins, and flavonoids in six crops (corn, sugarcane, coffee, canola, wheat, and tobacco) based on area and mass. The approach combines artificial intelligence, vegetation indices, wavelength selections, and remote sensing sensors to develop UV–VIS–NIR–SWIR models, contributing to advancements in digital agriculture. Our results demonstrate that PLSR, vegetation indexes, such as NDVI_750_, VOG, PSI, and PRI, despite extensive changes, and hyperspectral vegetation index (HVI) algorithm models exhibit precision and accuracy in calibration, cross-validation, and prediction. They provide comprehensive, robust, and rapid evaluation tools for crop quality and agronomic science. In addition, the use of full spectra based on hyperspectral curves has been demonstrated to provide a more precise classification and estimation of pigment profiles in C_3_ and C_4_ plant metabolisms compared to range spectra. Consequently, our study offers promising opportunities for the use of simultaneous algorithms in deciphering complex interactions between hyperspectral data and pigment profiling phenotyping in plants. This could lead to more accurate and precise spectral data classifications in the future, benefiting research in remote sensing for plant research.

## Figures and Tables

**Table 1:** Prediction of attributes for corn, sugarcane, coffee, canola, wheat, and tobacco crops using PLSR algorithms. Model fit was evaluated using R^2^, offset, RMSE, RPD, and bias parameters.

PLSR Models	Attributes	PLSR Parameters					
		r	R^2^	Offset	RMSE	RPD	Bias
**Predicted**	Chl*a* (mg m^−2^)	0.92	0.85	55.6	55.5	2.6	0.001
	Chl*b* (mg m^−2^)	0.92	0.84	57.1	57.1	2.5	0.001
	Chl*a*+*b* (mg m^−2^)	0.93	0.86	108.1	106.1	2.7	0.001
	Car (mg m^−2^)	0.94	0.89	16.9	17.0	3.0	0.001
	AnC (nmol m^−2^)	0.89	0.79	0.3	0.3	2.2	0.001
	Flv (nmol m^−2^)	0.92	0.85	10.9	10.8	2.5	1.59
	Chl*a* (mg g^−1^)	0.94	0.88	2.6	2.6	2.8	0.001
	Chl*b* (mg g^−1^)	0.89	0.79	1.5	1.5	2.2	0.001
	Chl*a*+*b* (mg g^−1^)	0.93	0.87	3.7	3.7	2.8	0.2
	Car (mg g^−1^)	0.93	0.86	0.8	0.8	2.7	0.01
	AnC (µmol g^−1^)	0.88	0.77	0.1	0.1	2.1	0.02
	Flv (µmol g^−1^)	0.93	0.87	2.2	2.2	2.8	0.32

## Data Availability

The data presented in this study are available in Supplementary Materials.

## Supplementary Materials

The following supporting information can be downloaded at: https://www.mdpi.com/article/10.3390/plants12122347/s1, Figure S1: (A) Correlation coefficients and (B) PC loadings in the 350–2500 nm range, represented by light to dark purple lines for PC1, PC2, and PC3, respectively. The red line indicates the −0.70 to +0.70 correlation range, with dots marking the 700 and 1300 nm delimitations. Figure S2: Matrix of correlation displayed accept and error frequency by model-based UV–VIS–NIR–SWIR comprising reflectance data to corn, sugarcane, coffee, canola, wheat, and tobacco plants. (A) 350–700 nm; (B) 700–1300 nm; (C) 1300–2500 nm; (D) 350–2500 nm. The correlation model test (frequency number) indicated a maximum simulation to correct classification-based independent data (scale 0 at 100; light green to dark blue). Abbreviations for models are indicated in the Material and Methods section. Figure S3: β–loadings and weighted coefficients predicted PLSR method-based UV–VIS–NIR–SWIR hyperspectral data. (A) Chlorophyll *a* (Chl*a*; mg m^–2^); (B) Chlorophyll *b* (Chl*b*; mg m^–2^); (C) Chlorophyll *a*+*b* (Chl*a*+*b*; mg m^–2^); (D) Carotenoids (Car; mg m^–2^); (E) Anthocyanins (AnC; nmol cm^–2^); (F) Flavonoids (Flv; nmol cm^–2^); (G) Chlorophyll *a* (Chla; mg g^–1^); (H) Chlorophyll *b* (Chl*b*; mg g^–1^); (I) Chlorophyll *a*+*b* (Chl*a*+*b*; mg g^–1^); (J) Carotenoids (Car; mg g^–1^); (K) Anthocyanins (AnC; µmol g^–1^); (L) Flavonoids (Flv; µmol g^–1^). Blue line (β–loadings) and red line (weighted coefficients). Table S1: Vegetation indexes calculated from the hyperspectral reflectance of leaves. Table S2: Descriptive analysis of pigment attribute-based area and mass. (*n* = 360). Table S3: Simultaneous PLSR statistical models obtained by calibration and cross-validation phase by pigments for corn, sugarcane, coffee, canola, wheat, and tobacco crops. Model goodness-of-fit (R^2^), offset, root mean square error (RMSE), ratio of performance to deviation (RPD), and bias parameters from UV–VIS–NIR–SWIR hyperspectral data. Abbreviation for attributes are indicated in the Material and Methods section. References [52,53,54,55,56,57,58,59,60,61,62] are cited in the Supplementary Materials.

## References

[1] Boshkovski B., Doupis G., Zapolska A., Kalaitzidis C., Koubouris G. (2022). Hyperspectral Imagery Detects Water Deficit and Salinity Effects on Photosynthesis and Antioxidant Enzyme Activity of Three Greek Olive Varieties. Sustainability.

[2] Zhang N., Zhou X., Kang M., Hu B.-G., Heuvelink E., Marcelis L.F.M. (2022). Machine Learning versus Crop Growth Models: An Ally, Not a Rival. AoB Plants.

[3] El-Hendawy S., Al-Suhaibani N., Mubushar M., Tahir M.U., Marey S., Refay Y., Tola E. (2022). Combining Hyperspectral Reflectance and Multivariate Regression Models to Estimate Plant Biomass of Advanced Spring Wheat Lines in Diverse Phenological Stages under Salinity Conditions. Appl. Sci..

[4] Fu Y., Yang G., Song X., Li Z., Xu X., Feng H., Zhao C. (2021). Improved Estimation of Winter Wheat Aboveground Biomass Using Multiscale Textures Extracted from UAV-Based Digital Images and Hyperspectral Feature Analysis. Remote Sens..

[5] Yoosefzadeh-Najafabadi M., Tulpan D., Eskandari M. (2021). Using Hybrid Artificial Intelligence and Evolutionary Optimization Algorithms for Estimating Soybean Yield and Fresh Biomass Using Hyperspectral Vegetation Indices. Remote Sens..

[6] Li K.-Y., de Lima R., Burnside N.G., Vahtmäe E., Kutser T., Sepp K., Cabral Pinheiro V.H., Yang M.-D., Vain A., Sepp K. (2022). Toward Automated Machine Learning-Based Hyperspectral Image Analysis in Crop Yield and Biomass Estimation. Remote Sens..

[7] Wang D., Cao W., Zhang F., Li Z., Xu S., Wu X. (2022). A Review of Deep Learning in Multiscale Agricultural Sensing. Remote Sens..

[8] Mao Y., Li H., Wang Y., Fan K., Song Y., Han X., Zhang J., Ding S., Song D., Wang H. (2022). Prediction of Tea Polyphenols, Free Amino Acids and Caffeine Content in Tea Leaves during Wilting and Fermentation Using Hyperspectral Imaging. Foods.

[9] SharathKumar M., Heuvelink E., Marcelis L.F.M. (2020). Vertical Farming: Moving from Genetic to Environmental Modification. Trends Plant Sci..

[10] Clemente A.A., Maciel G.M., Siquieroli A.C.S., de Araujo Gallis R.B., Pereira L.M., Duarte J.G. (2021). High-Throughput Phenotyping to Detect Anthocyanins, Chlorophylls, and Carotenoids in Red Lettuce Germplasm. Int. J. Appl. Earth Obs. Geoinf..

[11] Cotrozzi L., Lorenzini G., Nali C., Pellegrini E., Saponaro V., Hoshika Y., Arab L., Rennenberg H., Paoletti E. (2020). Hyperspectral Reflectance of Light-Adapted Leaves Can Predict Both Dark- and Light-Adapted Chl Fluorescence Parameters, and the Effects of Chronic Ozone Exposure on Date Palm (*Phoenix dactylifera*). Int. J. Mol. Sci..

[12] El-Sharkawy M., Sheta A., El-Wahed M., Arafat S., Behiery O. (2016). Precision Agriculture Using Remote Sensing and GIS for Peanut Crop Production in Arid Land. Int. J. Plant Soil Sci..

[13] Falcioni R., Gonçalves J.V.F., de Oliveira K.M., Antunes W.C., Nanni M.R. (2022). VIS-NIR-SWIR Hyperspectroscopy Combined with Data Mining and Machine Learning for Classification of Predicted Chemometrics of Green Lettuce. Remote Sens..

[14] Falcioni R., Gonçalves J.V.F., de Oliveira K.M., de Oliveira C.A., Demattê J.A.M., Antunes W.C., Nanni M.R. (2023). Enhancing Pigment Phenotyping and Classification in Lettuce through the Integration of Reflectance Spectroscopy and AI Algorithms. Plants.

[15] Falcioni R., Antunes W.C., Demattê J.A.M., Nanni M.R. (2023). Biophysical, Biochemical, and Photochemical Analyses Using Reflectance Hyperspectroscopy and Chlorophyll a Fluorescence Kinetics in Variegated Leaves. Biology.

[16] Crusiol L.G.T., Sun L., Sun Z., Chen R., Wu Y., Ma J., Song C. (2022). In-Season Monitoring of Maize Leaf Water Content Using Ground-Based and UAV-Based Hyperspectral Data. Sustainability.

[17] Fernandes A.M., Fortini E.A., Müller L.A.D.C., Batista D.S., Vieira L.M., Silva P.O., do Amaral C.H., Poethig R.S., Otoni W.C. (2020). Leaf Development Stages and Ontogenetic Changes in Passionfruit (*Passiflora edulis* Sims.) Are Detected by Narrowband Spectral Signal. J. Photochem. Photobiol. B Biol..

[18] Silva C.A., Nanni M.R., Teodoro P.E., Silva G.F.C. (2017). Vegetation Indices for Discrimination of Soybean Areas: A New Approach. Agron. J..

[19] Wang Y., Ma H., Wang J., Liu L., Pietikäinen M., Zhang Z., Chen X. (2021). Hyperspectral Monitor of Soil Chromium Contaminant Based on Deep Learning Network Model in the Eastern Junggar Coalfield. Spectrochim. Acta Part A Mol. Biomol. Spectrosc..

[20] Hassanzadeh A., Murphy S.P., Pethybridge S.J., van Aardt J. (2020). Growth Stage Classification and Harvest Scheduling of Snap Bean Using Hyperspectral Sensing: A Greenhouse Study. Remote Sens..

[21] Feng L., Zhang Z., Ma Y., Du Q., Williams P., Drewry J., Luck B. (2020). Alfalfa Yield Prediction Using UAV-Based Hyperspectral Imagery and Ensemble Learning. Remote Sens..

[22] Ropelewska E. (2022). Application of Imaging and Artificial Intelligence for Quality Monitoring of Stored Black Currant (*Ribes nigrum* L.). Foods.

[23] Crusiol L.G.T., Nanni M.R., Furlanetto R.H., Sibaldelli R.N.R., Sun L., Gonçalves S.L., Foloni J.S.S., Mertz-Henning L.M., Nepomuceno A.L., Neumaier N. (2023). Assessing the Sensitive Spectral Bands for Soybean Water Status Monitoring and Soil Moisture Prediction Using Leaf-Based Hyperspectral Reflectance. Agric. Water Manag..

[24] Phuangsaijai N., Theanjumpol P., Kittiwachana S. (2022). Performance Optimization of a Developed Near-Infrared Spectrometer Using Calibration Transfer with a Variety of Transfer Samples for Geographical Origin Identification of Coffee Beans. Molecules.

[25] Wang H., Mortensen A.K., Mao P., Boelt B., Gislum R. (2019). Estimating the Nitrogen Nutrition Index in Grass Seed Crops Using a UAV-Mounted Multispectral Camera. Int. J. Remote Sens..

[26] Falcioni R., Moriwaki T., Gibin M.S., Vollmann A., Pattaro M.C., Giacomelli M.E., Sato F., Nanni M.R., Antunes W.C. (2022). Classification and Prediction by Pigment Content in Lettuce (*Lactuca sativa* L.) Varieties Using Machine Learning and ATR-FTIR Spectroscopy. Plants.

[27] Izenman A.J. (2008). Modern Multivariate Statistical Techniques.

[28] Franca T., Goncalves D., Cena C. (2022). ATR-FTIR Spectroscopy Combined with Machine Learning for Classification of PVA/PVP Blends in Low Concentration. Vib. Spectrosc..

[29] Braga P., Crusiol L.G.T., Nanni M.R., Caranhato A.L.H., Fuhrmann M.B., Nepomuceno A.L., Neumaier N., Farias J.R.B., Koltun A., Gonçalves L.S.A. (2021). Vegetation Indices and NIR-SWIR Spectral Bands as a Phenotyping Tool for Water Status Determination in Soybean. Precis. Agric..

[30] Sobejano-Paz V., Mikkelsen T.N., Baum A., Mo X., Liu S., Köppl C.J., Johnson M.S., Gulyas L., García M. (2020). Hyperspectral and Thermal Sensing of Stomatal Conductance, Transpiration, and Photosynthesis for Soybean and Maize under Drought. Remote Sens..

[31] Yang X., Xu H., Shao L., Li T., Wang Y., Wang R. (2018). Response of Photosynthetic Capacity of Tomato Leaves to Different LED Light Wavelength. Environ. Exp. Bot..

[32] Matysiak B., Ropelewska E., Wrzodak A., Kowalski A., Kaniszewski S. (2022). Yield and Quality of Romaine Lettuce at Different Daily Light Integral in an Indoor Controlled Environment. Agronomy.

[33] Huerta R.R., Saldaña M.D.A. (2018). Pressurized Fluid Treatment of Barley and Canola Straws to Obtain Carbohydrates and Phenolics. J. Supercrit. Fluids.

[34] Fan K., Li F., Chen X., Li Z., Mulla D.J. (2022). Nitrogen Balance Index Prediction of Winter Wheat by Canopy Hyperspectral Transformation and Machine Learning. Remote Sens..

[35] da Silva Junior C.A., Nanni M.R., Shakir M., Teodoro P.E., de Oliveira-Júnior J.F., Cezar E., de Gois G., Lima M., Wojciechowski J.C., Shiratsuchi L.S. (2018). Soybean Varieties Discrimination Using Non-Imaging Hyperspectral Sensor. Infrared Phys. Technol..

[36] Guo T., Tan C., Li Q., Cui G., Li H. (2019). Estimating Leaf Chlorophyll Content in Tobacco Based on Various Canopy Hyperspectral Parameters. J. Ambient Intell. Humaniz. Comput..

[37] Zhang H., Zhang L., Wang S., Zhang L. (2022). Online Water Quality Monitoring Based on UV–Vis Spectrometry and Artificial Neural Networks in a River Confluence near Sherfield-on-Loddon. Environ. Monit. Assess..

[38] Zhou Q., Yu L., Zhang X., Liu Y., Zhan Z., Ren L., Luo Y. (2022). Fusion of UAV Hyperspectral Imaging and LiDAR for the Early Detection of EAB Stress in Ash and a New EAB Detection Index NDVI(776,678). Remote Sens..

[39] Giordano M., El-Nakhel C., Carillo P., Colla G., Graziani G., Di Mola I., Mori M., Kyriacou M.C., Rouphael Y., Soteriou G.A. (2022). Plant-Derived Biostimulants Differentially Modulate Primary and Secondary Metabolites and Improve the Yield Potential of Red and Green Lettuce Cultivars. Agronomy.

[40] Shi M., Gu J., Wu H., Rauf A., Bin Emran T., Khan Z., Mitra S., Aljohani A.S.M., Alhumaydhi F.A., Al-Awthan Y.S. (2022). Phytochemicals, Nutrition, Metabolism, Bioavailability, and Health Benefits in Lettuce: A Comprehensive Review. Antioxidants.

[41] Wang L., Chang Q., Li F., Yan L., Huang Y., Wang Q., Luo L. (2019). Effects of Growth Stage Development on Paddy Rice Leaf Area Index Prediction Models. Remote Sens..

[42] Jin J., Wang Q. (2019). Selection of Informative Spectral Bands for PLS Models to Estimate Foliar Chlorophyll Content Using Hyperspectral Reflectance. IEEE Trans. Geosci. Remote Sens..

[43] Guardado Yordi E., Koelig R., Matos M.J., Pérez Martínez A., Caballero Y., Santana L., Pérez Quintana M., Molina E., Uriarte E. (2019). Artificial Intelligence Applied to Flavonoid Data in Food Matrices. Foods.

[44] Cezar E., Nanni M.R., Guerrero C., da Silva Junior C.A., Cruciol L.G.T., Chicati M.L., Silva G.F.C. (2019). Organic Matter and Sand Estimates by Spectroradiometry: Strategies for the Development of Models with Applicability at a Local Scale. Geoderma.

[45] Koh J.C.O., Banerjee B.P., Spangenberg G., Kant S. (2022). Automated Hyperspectral Vegetation Index Derivation Using a Hyperparameter Optimisation Framework for High-Throughput Plant Phenotyping. New Phytol..

[46] Rodrigues M., Berti de Oliveira R., Leboso Alemparte Abrantes dos Santos G., Mayara de Oliveira K., Silveira Reis A., Herrig Furlanetto R., Antônio Yanes Bernardo Júnior L., Silva Coelho F., Rafael Nanni M. (2022). Rapid Quantification of Alkaloids, Sugar and Yield of Tobacco (*Nicotiana tabacum* L.) Varieties by Using Vis–NIR–SWIR Spectroradiometry. Spectrochim. Acta Part A Mol. Biomol. Spectrosc..

[47] Kior A., Sukhov V., Sukhova E. (2021). Application of Reflectance Indices for Remote Sensing of Plants and Revealing Actions of Stressors. Photonics.

[48] Ferri C.P., Formaggio A.R., Schiavinato M.A. (2004). Narrow Band Spectral Indexes for Chlorophyll Determination in Soybean Canopies [*Glycine max* (L.) Merril]. Braz. J. Plant Physiol..

[49] Jin J., Huang N., Huang Y., Yan Y., Zhao X., Wu M. (2022). Proximal Remote Sensing-Based Vegetation Indices for Monitoring Mango Tree Stem Sap Flux Density. Remote Sens..

[50] Ryu J.H., Jeong H., Cho J. (2020). Performances of Vegetation Indices on Paddy Rice at Elevated Air Temperature, Heat Stress, and Herbicide Damage. Remote Sens..

[51] Crusiol L.G.T., Sun L., Sibaldelli R.N.R., Junior V.F., Furlaneti W.X., Chen R., Sun Z., Wuyun D., Chen Z., Nanni M.R. (2022). Strategies for Monitoring Within-Field Soybean Yield Using Sentinel-2 Vis-NIR-SWIR Spectral Bands and Machine Learning Regression Methods. Precis. Agric..

[52] Gitelson A., Merzlyak M.N. (1994). Spectral Reflectance Changes Associated with Autumn Senescence of *Aesculus hippocastanum* L. and *Acer platanoides* L. Leaves. Spectral Features and Relation to Chlorophyll Estimation. J. Plant Physiol..

[53] Stimson H.C., Breshears D.D., Ustin S.L., Kefauver S.C. (2005). Spectral Sensing of Foliar Water Conditions in Two Co-Occurring Conifer Species: *Pinus edulis* and *Juniperus monosperma*. Remote Sens. Environ..

[54] Lichtenthaler H.K. (1996). Vegetation Stress: An Introduction to the Stress Concept in Plants. J. Plant Physiol..

[55] Chappelle E.W., Kim M.S., McMurtrey J.E. (1992). Ratio Analysis of Reflectance Spectra (RARS): An Algorithm for the Remote Estimation of the Concentrations of Chlorophyll A, Chlorophyll B, and Carotenoids in Soybean Leaves. Remote Sens. Environ..

[56] Gitelson A.A., Zur Y., Chivkunova O.B., Merzlyak M.N. (2002). Assessing Carotenoid Content in Plant Leaves with Reflectance Spectroscopy. Photochem. Photobiol..

[57] Blackburn G.A. (1998). Spectral Indices for Estimating Photosynthetic Pigment Concentrations: A Test Using Senescent Tree Leaves. Int. J. Remote Sens..

[58] Merzlyak M.N., Gitelson A.A., Chivkunova O.B., Rakitin V.Y.U. (1999). Non-Destructive Optical Detection of Pigment Changes during Leaf Senescence and Fruit Ripening. Physiol. Plant..

[59] Vogelmann J.E., Rock B.N., Moss D.M. (1993). Red Edge Spectral Measurements from Sugar Maple Leaves. Int. J. Remote Sens..

[60] Hunt E.R., Rock B.N. (1989). Detection of Changes in Leaf Water Content Using Near- and Middle-Infrared Reflectances. Remote Sens. Environ..

[61] Peñuelas J., Filella I., Gamon J.A. (1995). Assessment of Photosynthetic Radiation-Use Efficiency with Spectral Reflectance. New Phytol..

[62] Metternicht G. (2003). Vegetation Indices Derived from High-Resolution Airborne Videography for Precision Crop Management. Int. J. Remote Sens..

