# Supplementary material for: Reflectance Spectroscopy for the Classification and Prediction of Pigments in Agronomic Crops
Source: Plants (Basel). 2023 Jun 16;12(12):2347. doi: 10.3390/plants12122347 (PMC10304803; doi:10.3390/plants12122347)
Supplement: Supplementary file 1 [file plants-12-02347-s001.zip › plants-2442385-SI.pdf]

plants

Supplementary files

Reflectance Spectroscopy for the Classification and Prediction of Pigments in Agronomic Crops

Renan Falcioni [1,*], Werner Camargos Antunes [1], José Alexandre M. Demattê [2] and Marcos Rafael Nanni [1]

[1] Graduate Program in Agronomy, Department of Agronomy, State University of Maringá, Av. Colombo, 5790, Maringá 87020-900, PR, Brazil; wcantunes@uem.br (W.C.A.); mrnanni@uem.br (M.R.N.)
[2] Department of Soil Science, Luiz de Queiroz College of Agriculture, University of São Paulo, Av. Pádua Dias, 11, Piracicaba 13418-260, SP, Brazil; jamdemat@usp.br
* Correspondence: renanfalcioni@gmail.com; Tel.: +55-44-3011-8940

Table S1. Vegetation indexes calculated from the hyperspectral reflectance of leaves.

Index	Equation	Reference
$NDVI_{750}$ = Normalized Difference Vegetation Index ϱ750	$(R_{750}–R_{705})/(R_{750}+R_{705})$	[52]
WBI = Water Band Index	$(R_{900})/(R_{970})$	[53]
RARS = Ratio Analysis of Reflectance Spectra	$(R_{746})/(R_{513})$	[53]
ARI1 = Anthocyanin Reflectance Index	$(1/R_{550})–(1/R_{700})$	[54]
PSND = Pigment Specific Normalized Difference	$(R_{800}–R_{470})/(R_{800}+R_{470})$	[55]
SIPI = Structurally Insensitive Pigment Index	$(R_{800}–R_{445})/(R_{800}–R_{680})$	[55]
PSRI = Plant Senescence Reflectance Index	$(R_{680}–R_{500})/(R_{750})$	[56]
PSRI2 = Plant Senescence Reflectance Index 2	$(R_{672})/(R_{550}+R_{708})$	[56]
PSSRc = Pigment–specific Simple Ratio	$(R_{800})/(R_{500})$	[55]
VOG1 = Vogelmann Index 1	$(R_{740})/(R_{720})$	[57]
VOG2 = Vogelmann Index 2	$(R_{734}–R_{747})/(R_{715}+R_{726})$	[58]
MSI = Moisture Stress Index	(R_{1650}/R_{830})	[59]
PRI = Photochemical Reflectance Index	$(R_{530}–R_{570})/(R_{530}+R_{570})$	[60]
PVR = Normalized Difference Photosynthetic	$(R_{550}–R_{650})/(R_{550}+R_{650})$	[61]
FR = Fluorescence Ratio	$(R_{690})/(R_{740})$	[62]

Table S2. Descriptive analysis of pigment attribute−based area and mass. (n = 360).

Attributes	Count (n)	Mean	Median	Minimum	Maximum	CV (%)
Chl*a* (mg m^{-2})	360	330.6	329.6	136.9	713.0	42.2
Chl*b* (mg m^{-2})	360	210.1	154.2	61.0	994.4	69.7
Chl*a*+*b* (mg m^{-2})	360	540.7	475.5	197.9	1707.4	51.1
Car (mg m^{-2})	360	122.3	116.6	52.4	287.8	41.3
AnC (nmol m^{-2})	360	0.9	0.8	0.1	2.1	65.2
Flv (nmol m^{-2})	360	73.4	68.9	15.8	116.8	37.1
Chl*a* (mg g^{-1})	360	7.0	6.0	1.9	29.6	73.8
Chl*b* (mg g^{-1})	360	4.1	3.5	1.0	14.1	62.6
Chl*a*+*b* (mg g^{-1})	360	11.1	9.7	3.1	41.1	66.6
Car (mg g^{-1})	360	2.5	2.3	0.7	9.4	61.3
AnC (μmol g^{-1})	360	0.2	0.1	0.0	0.7	93.4
Flv (μmol g^{-1})	360	11.9	11.3	3.5	20.4	33.4

Table S3. Simultaneous PLSR statistical models obtained by calibration and cross–validation phase by pigments for corn, sugarcane, coffee, canola, wheat, and tobacco crops. Model goodness–of–fit (R^2), offset, root mean square error (RMSE), ratio of performance to deviation (RPD), and bias parameters from UV–VIS–NIR–SWIR hyperspectral data. Abbreviation for attributes were indicate material and methods sections.

PLSR Models	Atributes	PLSR Parameters					
		r	R^2	Offset	RMSE	RPD	Bias
Calibration	Chl*a* (mg m^{-2})	0.92	0.85	48.8	54.3	2.6	−
	Chl*b* (mg m^{-2})	0.91	0.83	33.3	51.3	2.4	−
	Chl*a*+*b* (mg m^{-2})	0.90	0.81	97.7	111.7	2.3	−
	Car (mg m^{-2})	0.93	0.87	16.0	18.2	2.7	−
	AnC (nmol m^{-2})	0.93	0.87	0.1	0.2	2.8	−
	Flv (nmol m^{-2})	0.92	0.84	11.6	10.5	2.5	−
	Chl*a* (mg g^{-1})	0.90	0.81	1.4	2.4	2.3	−
	Chl*b* (mg g^{-1})	0.88	0.78	1.3	1.4	2.1	−
	Chl*a*+*b* (mg g^{-1})	0.88	0.77	2.6	3.6	2.1	−
	Car (mg g^{-1})	0.89	0.79	0.5	0.8	2.2	−
	AnC (μmol g^{-1})	0.94	0.89	0.0	0.1	3.0	−
	Flv (μmol g^{-1})	0.89	0.80	2.4	1.7	2.2	−
Cross−Validation	Chl*a* (mg m^{-2})	0.92	0.85	50.3	56.8	2.5	−
	Chl*b* (mg m^{-2})	0.90	0.82	34.8	54.2	2.3	−
	Chl*a*+*b* (mg m^{-2})	0.90	0.81	99.9	114.4	2.3	−
	Car (mg m^{-2})	0.93	0.86	16.0	18.8	2.7	−
	AnC (nmol m^{-2})	0.93	0.87	0.1	0.2	2.7	−
	Flv (nmol m^{-2})	0.92	0.85	12.2	10.9	2.6	−
	Chl*a* (mg g^{-1})	0.88	0.78	1.6	2.5	2.1	−
	Chl*b* (mg g^{-1})	0.88	0.78	1.4	1.5	2.1	−
	Chl*a*+*b* (mg g^{-1})	0.88	0.77	2.6	3.8	2.1	−
	Car (mg g^{-1})	0.88	0.77	0.6	0.7	2.1	−
	AnC (μmol g^{-1})	0.94	0.88	0.0	0.0	2.9	−
	Flv (μmol g^{-1})	0.89	0.79	2.5	1.7	2.2	−

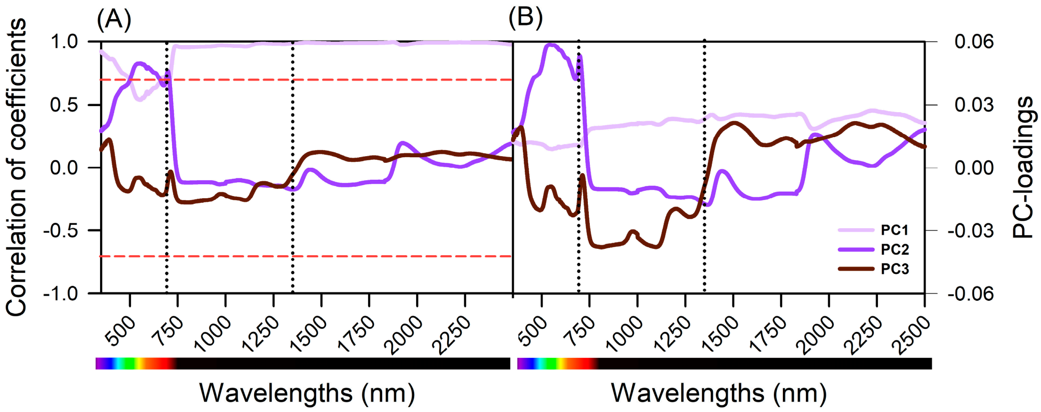

Figure S1. (A) Correlation coefficients and **(B)** PC loadings in the 350–2500 nm range, represented by light to dark purple lines for PC1, PC2, and PC3, respectively. The red line indicates the −0.70 to +0.70 correlation range, with dots marking the 700 and 1300 nm delimitations.

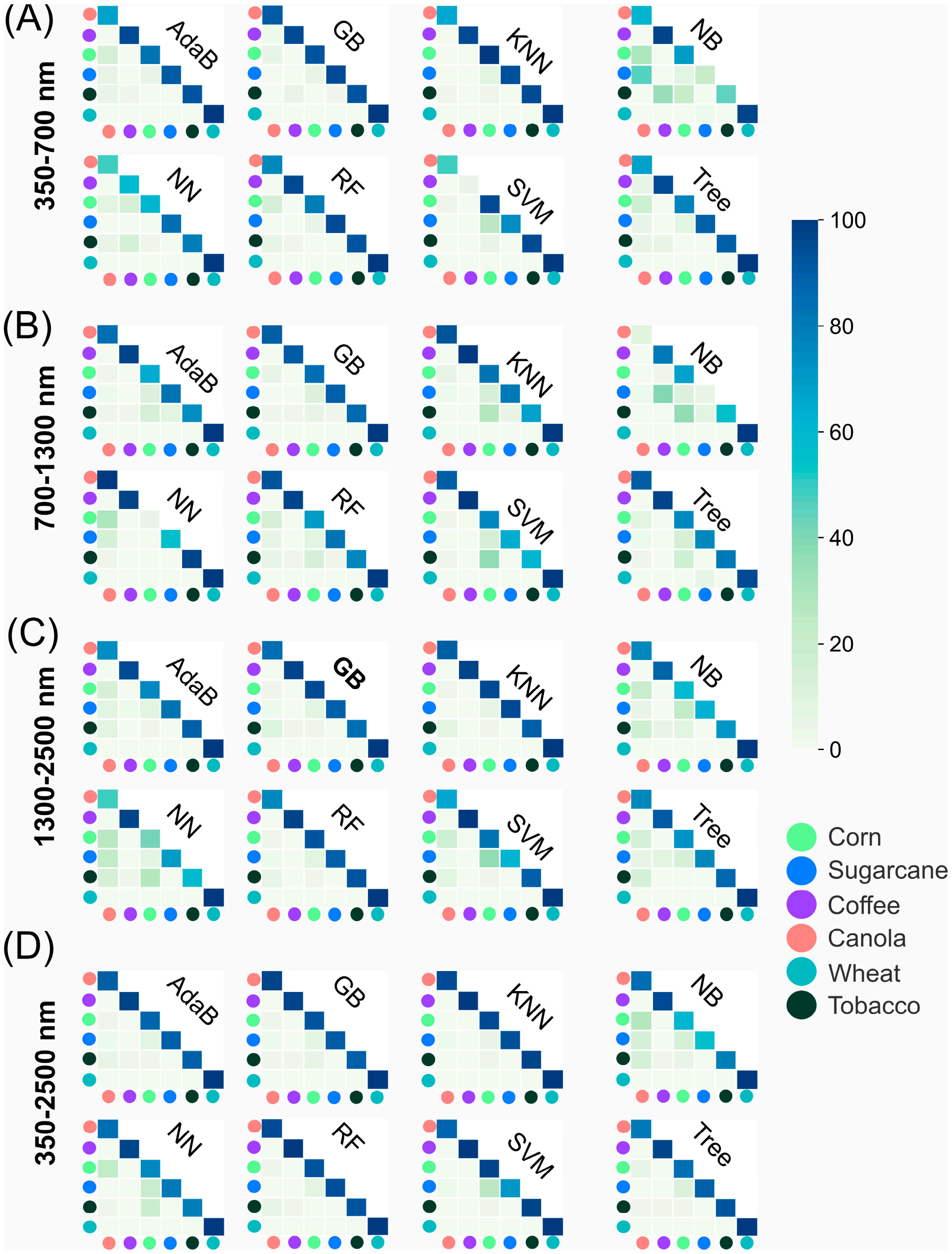

Figure S2. Matrix of correlation displayed accept and error frequency by model–based UV–VIS–NIR–SWIR comprising reflectance data to corn, sugarcane, coffee, canola, wheat, and tobacco plants. **(A)** 350–700 nm; **(B)** 700–1300 nm; **(C)** 1300–2500 nm); **(D)** 350–2500 nm. The correlation model test (frequency number) indicated a maximum simulation to correct classification–based independent data (scale 0 at 100; light green to dark blue). Abbreviation for models were indicate material and methods sections.

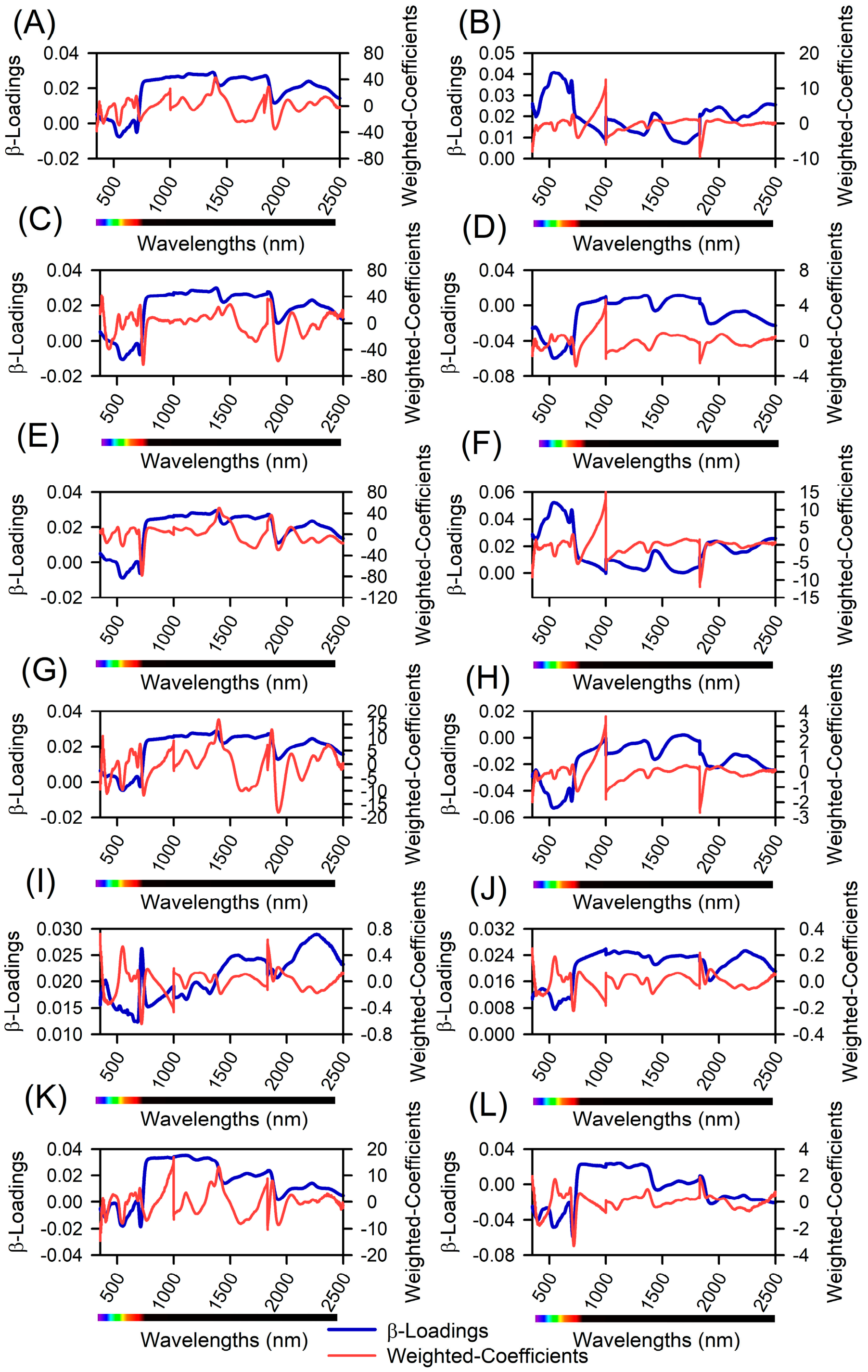

Figure S3. β–loadings and weighted–coefficients predicted PLSR method–based UV–VIS–NIR–SWIR hyperspectral data. **(A)** Chlorophyll *a* (Chl*a*; mg m^{-2}); **(B)** Chlorophyll *b* (Chl*b*; mg m^{-2}); **(C)** Chlorophyll *a+b* (Chl*a+b*; mg m^{-2}); **(D)** Carotenoids (Car; mg m^{-2}); **(E)** Anthocyanins (AnC; nmol cm^{-2}); **(F)** Flavonoids (Flv; nmol cm^{-2}); **(G)** Chlorophyll *a* (Chl*a*; mg g^{-1}); **(H)** Chlorophyll *b* (Chl*b*; mg g^{-1}); **(I)** Chlorophyll *a+b* (Chl*a+b*; mg g^{-1}); **(J)** Carotenoids (Car; mg g^{-1}); **(K)** Anthocyanins (AnC; μmol g^{-1}); **(L)** Flavonoids (Flv; μmol g^{-1}). Blue line (β–loadings) and red line (weighted–coefficients).

References

52. Gitelson, A.; Merzlyak, M.N. Spectral Reflectance Changes Associated with Autumn Senescence of *Aesculus hippocastanum* L. and *Acer platanoides* L. Leaves. Spectral Features and Relation to Chlorophyll Estimation. *J. Plant Physiol.* **1994**, 143, 286–292.
53. Stimson, H.C.; Breshears, D.D.; Ustin, S.L.; Kefauver, S.C. Spectral Sensing of Foliar Water Conditions in Two Co-Occurring Conifer Species: *Pinus edulis* and *Juniperus monosperma*. *Remote Sens. Environ.* **2005**, 96, 108–118.
54. Lichtenthaler, H.K. Vegetation Stress: An Introduction to the Stress Concept in Plants. *J. Plant Physiol.* **1996**, 148, 4–14.
55. Chappelle, E.W.; Kim, M.S.; McMurtrey, J.E. Ratio Analysis of Reflectance Spectra (RARS): An Algorithm for the Remote Estimation of the Concentrations of Chlorophyll A, Chlorophyll B, and Carotenoids in Soybean Leaves. *Remote Sens. Environ.* **1992**, 39, 239–247.
56. Gitelson, A.A.; Zur, Y.; Chivkunova, O.B.; Merzlyak, M.N. Assessing Carotenoid Content in Plant Leaves with Reflectance Spectroscopy. *Photochem. Photobiol.* **2002**, 75, 272.
57. Blackburn, G.A. Spectral Indices for Estimating Photosynthetic Pigment Concentrations: A Test Using Senescent Tree Leaves. *Int. J. Remote Sens.* **1998**, 19, 657–675.
58. Merzlyak, M.N.; Gitelson, A.A.; Chivkunova, O.B.; Rakitin, V.Y.U. Non-Destructive Optical Detection of Pigment Changes during Leaf Senescence and Fruit Ripening. *Physiol. Plant.* **1999**, 106, 135–141.
59. Vogelmann, J.E.; Rock, B.N.; Moss, D.M. Red Edge Spectral Measurements from Sugar Maple Leaves. *Int. J. Remote Sens.* **1993**, 14, 1563–1575.
60. Hunt, E.R.; Rock, B.N. Detection of Changes in Leaf Water Content Using Near- and Middle-Infrared Reflectances. *Remote Sens. Environ.* **1989**, 30, 43–54.
61. Peñuelas, J.; Filella, I.; Gamon, J.A. Assessment of Photosynthetic Radiation-Use Efficiency with Spectral Reflectance. *New Phytol.* **1995**, 131, 291–296.
62. Metternicht, G. Vegetation Indices Derived from High-Resolution Airborne Videography for Precision Crop Management. *Int. J. Remote Sens.* **2003**, 24, 2855–2877.
